# Reference Values for the Six-Minute Walk Test in Healthy Children and Adolescents: a Systematic Review

**DOI:** 10.5935/1678-9741.20160081

**Published:** 2016

**Authors:** Lucas de Assis Pereira Cacau, Valter Joviniano de Santana-Filho, Luana G. Maynard, Mansueto Gomes Neto, Marcelo Fernandes, Vitor Oliveira Carvalho

**Affiliations:** 1 Departamento de Fisioterapia e Pós-Graduação em Ciências da Saúde da Universidade Federal de Sergipe (UFS), Aracaju, SE, Brazil.; 2 The GREAT Group (Grupo de Estudos em Atividade Física), Brazil.; 3 Departamento de Fisioterapia da Universidade Tiradentes, (Unit), Aracaju, SE, Brazil.; 4 Departamento de Fisioterapia da Universidade Federal da Bahia (UFBA), Salvador, BA, Brazil.; 5 Departamento de Fisioterapia da Universidade Presbiteriana Mackenzie, São Paulo, SP, Brazil.

**Keywords:** Cardiopulmonary Bypass, Adolescent, Cardiology, Child Health

## Abstract

**Objective:**

The aim of the study is to compare the available reference values and the six-minute walk test equations in healthy children/adolescents. Our systematic review was planned and performed in accordance with the PRISMA guidelines. We included all studies that established reference values for the six-minute walk test in healthy children/adolescents.

**Methods:**

To perform this review, a research was performed in PubMed, EMBASE (via SCOPUS) and Cochrane (LILACS), Bibliographic Index Spanish in Health Sciences, Organization Collection Pan-American Health Organization, Publications of the World Health Organization and Scientific Electronic Library Online (SciELO) via Virtual Health Library until June 2015 without language restriction.

**Results:**

The initial research identified 276 abstracts. Twelve studies met the inclusion criteria and were fully reviewed and approved by both reviewers. None of the selected studies presented sample size calculation. Most of the studies recruited children and adolescents from school. Six studies reported the use of random samples. Most studies used a corridor of 30 meters. All studies followed the American Thoracic Society guidelines to perform the six-minute walk test. The walked distance ranged 159 meters among the studies. Of the 12 included studies, 7 (58%) reported descriptive data and 6 (50%) established reference equation for the walked distance in the six-minute walk test.

**Conclusion:**

The reference value for the six-minute walk test in children and adolescents ranged substantially from studies in different countries. A reference equation was not provided in all studies, but the ones available took into account well established variables in the context of exercise performance, such as height, heart rate, age and weight. Countries that did not established reference values for the six-minute walk test should be encouraged to do because it would help their clinicians and researchers have a more precise interpretation of the test.

**Table:** 

Abbreviations, acronyms & symbols	
**6MWT**	**= Six-minute walk test**
**ATS**	**= American Thoracic Society**
**FAPITEC/SE**	**= Fundação de Apoio à Pesquisa e à Inovação**
	**Tecnológica do Estado de Sergipe, Brasil**
**IBECS**	**= Spanish Bibliographic Index on Health Sciences**
**LILACS**	**= Latin American and Caribbean Health Sciences**
**PAHO**	**= Collection of the Pan American Health Organization**
**PRISMA**	**= Preferred Reporting Items for Systematic Reviews**
	**and Meta-Analyses**
**SciELO**	**= Scientific Electronic Library Online**
**VHL**	**= Virtual Health Library**
**WHO**	**= World Health Organization**

## INTRODUCTION

The six-minute walk test (6MWT) is a functional test conceptually performed in a submaximal effort, which has been proposed to reflect activities of daily living^[1]^. Since the development of the 6MWT in the early 1970s^[2]^, this test is growing its importance in clinical practice and research. This exercise test is enshrined in patients with several cardiopulmonary and metabolic disorders, such as chronic obstructive pulmonary disease, exercise tolerance in severely ill children, postoperative cardiac surgery, congenital heart disease and as predicted mortality in patients with heart failure^[3-8]^.

The 6MWT is growing its importance in clinical practice and in scientific context because it is of easy implementation, low cost and the maximal walked distance represents high prognostic value in several cardiopulmonary disorders^[3,4]^. This test is also widely used to assess exercise capacity before and after an intervention, such as an exercise-training program^[2]^. Briefly, patients are instructed to walk both ways for six minutes on a corridor around 30 meters, which is delimited by two cones. The maximum walked distance is the main outcome in the 6MWT^[2]^.

Although the 6MWT has been widely used in adults, its use in children and adolescents only increased significantly in the scientific literature over the past decade. In health children, the 6MWT has been proposed to be a reliable and valid functional test for assessing exercise tolerance^[7]^. Up to the present moment the literature brings the use of the 6MWT in children/adolescents with, congenital heart disease^[6]^, severe cardiac impairment (pre cardiac transplantation or pulmonary)^[8]^, cardiovascular disease, atherosclerosis, hypertension, and obesity in youth^[9]^, asthma^[10]^, cystic fibrosis^[11]^, end-stage renal disease^[12]^ and pulmonary hypertension^[13]^.

Measuring pretransplant 6-MWT tests for pediatric patients is valuable in predicting peri-operative outcomes after lung transplantation.

Considering the worldwide interest in the 6MWT, many countries already have established reference values for their children/adolescents. Moreover, it is not uncommon that clinicians and researchers from a country use a foreign reference value for the 6MWT. In this context, reference values are crucial to a correct interpretation of the test in clinical practice and scientific field^[14-25]^.

The aim of this report was to perform a systematic review of the reference values and equations for the 6MWT in healthy children/adolescents published in the literature. Our hypothesis is that the published reference values for the 6MWT can be different between countries, what deserves some attention from clinicians and researchers.

## METHODS

This systematic review was performed in accordance with the Preferred Reporting Items for Systematic Reviews and Meta-Analyses (PRISMA) Statement^[26]^.

### Eligibility Criteria

This systematic review was planned to include all studies that established reference values for the 6MWT in healthy children/adolescents. Studies were considered for inclusion regardless of language or size. Studies enrolling health children (from 4 to less than 12 years) and adolescents (from 12 to 18 years old) were included in this review^[22]^. We excluded studies: 1) that enrolled adults; 2) with unclear description of the population; 3) that used any equipment to incentive, assistors motivate the participants; and 4) that enrolled participants with any musculoskeletal, neurological, cardiovascular or respiratory disorders.

### Outcome of Interest

The main outcomes of interest were the reference value and the reference equation for the walked distance in the 6MWT established in different countries.

### Research Strategy

We did a researched on PubMed, EMBASE (via SCOPUS), and COCHRANE, Latin American and Caribbean Health Sciences (LILACS), Spanish Bibliographic Index on Health Sciences (IBECS), Collection of the Pan American Health Organization (PAHO), Publications from the World Health Organization (WHO, WHOLIS) and Scientific Electronic Library Online (SciELO) via Virtual Health Library (VHL) until June 2015 without language restriction. A standard protocol was set and, whenever possible, a standardized vocabulary was used. The following terms were used in our research: "walk test", "children", "reference", "adolescent". We reviewed the reference list of the included studies in order to detect other potentially eligible studies.

### Data Collection and Analysis

The research strategy was used to obtain titles and abstracts that might be relevant for our review. Two reviewers independently checked each title and abstract. If at least one of the reviewers considered one reference eligible, the full text was provided. Two reviewers also evaluated the full text articles and filled inclusion and exclusion criteria in a standard form. The reviewers discussed disagreements and a final decision was made by a third one^[27]^.

Two authors independently extracted data using standard data extraction forms, considering: 1) aspects of the study population, such as age, body mass index and gender; 2) if the test circuit is in accordance to the American Thoracic Society (ATS) guidelines; 3) length of the corridor (meters); 4) instructions; 5) encouragement; 6) standardization; 7) average of the walked distance; 8) reference equation for the walked distance; 9) side effects; 10) number of tests performed. A third reviewer resolved disagreements. Any relevant information about the selected studies was requested by e-mail.

### Quality of the Studies and Risk of Bias

The risk of bias was assessed according to Standards for Reporting of Diagnostic Accuracy (STARD)^[28]^:

1. Distribution by sex and age of the study population;
2. Date of inclusion and follow-up period of the study;
3. Test standard reference suitability of the chosen gold standard, evaluating whether this does not lead to misclassification of disease status;
4. Technical aspects of testing;
5. To evaluate the degree of data loss (missing data);
6. Earnings original false and true-positive, false and truenegative. Eventually, this data can be estimated from sensitivity, specificity, and positive and negative values of endpoint or reference test;
7. Guidelines for the gold standard and to examine research in a clear and representative form of the disease in question;
8. The confidence intervals and the standard error for the examination of performance measures;
9. The number of evaluators and their training for the exam in question and the gold standard;
10. Review Bias Attendance: verify that the test results in the study were evaluated in a masked form for outcomes and other tests (independent interpretation);
11. Verification Bias Attendance: the reference test may have been performed preferably in patients with positive tests, which is more frequent when the tests considered the gold standard are invasive. In this case, the selection of patients to perform the gold standard test is not random;
12. If the reference test was applied to all patients. If the examination in research and the gold standard have not been applied to all patients, which is ideal to assess whether the choice of patients for the tests occurred randomly, reducing the chance of bias;
13. Clinical Spectrum Bias Presence: absence of clinical spectrum representation of the studied disease in the study population. Evaluate demographic and clinical data of patients, such as age, sex, race, clinical features, symptoms, disease stage, duration, and comorbidities. The prevalence of the condition in this population offers broader view of the spectrum, circumstances and potential generalization;
14. In screening tests, there may be over-diagnosis bias (when a disease that could evolve asymptomatically is detected), representing excess bias (for diseases that develop slowly progressive, making them more "show" for because of screening) and early detection bias (overestimate the effects of clinical benefit) (Table 1).

**Table 1:** Quality of the studies and risk of bias according to Standards for Reporting of Diagnostic Accuracy (STARD).

	1	2	3	4	5	6	7	8	9	10	11	12	13	14
D'Silva et al.^[25]^	**✓**	-	NA	**✓**	-	-	NA	-	**✓**	NA	NA	NA	NA	NA
Klepper & Muir^[14]^	**✓**	**✓**	NA	**✓**	**✓**	**✓**	NA	**✓**	**✓**	NA	NA	NA	NA	NA
Rhamanad & Alnegimshi^[15]^	**✓**	-	NA	**✓**	**✓**	-	NA	-	**✓**	NA	NA	NA	NA	NA
Tonklang et al.^[16]^	**✓**	**✓**	NA	**✓**	**✓**	**✓**	NA	**✓**	**✓**	NA	NA	NA	NA	NA
Li et al.^[17]^	**✓**	**✓**	NA	**✓**	**✓**	**✓**	NA	**✓**	**✓**	NA	NA	NA	NA	NA
Saad et al.^[18]^	**✓**	**✓**	NA	**✓**	**✓**	**✓**	NA	**✓**	**✓**	NA	NA	NA	NA	NA
Goemans et al.^[19]^	**✓**	**✓**	NA	**✓**	**✓**	**✓**	NA	**✓**	**✓**	NA	NA	NA	NA	NA
Lammers et al.^[20]^	**✓**	-	NA	**✓**	**✓**	**✓**	NA	**✓**	**✓**	NA	NA	NA	NA	NA
Ulrich et al.^[21]^	**✓**	-	NA	**✓**	**✓**	**✓**	NA	-	**✓**	NA	NA	NA	NA	NA
Prietnitz et al.^[22]^	**✓**	**✓**	NA	**✓**	**✓**	**✓**	NA	**✓**	**✓**	NA	NA	NA	NA	NA
Gatica et al.^[23]^	**✓**	**✓**	NA	**✓**	**✓**	**✓**	NA	**✓**	**✓**	NA	NA	NA	NA	NA
Kanburoglu et al.^[24]^	**✓**	-	NA	**✓**	**✓**	**✓**	NA	**✓**	**✓**	NA	NA	NA	NA	NA

NA = not available

## RESULTS

### Description of the Selected Studies

The initial research identified 276 abstracts, from which 30 studies were considered as potentially relevant and were considered for detailed analysis. Considering the analysis, 1 article was a review; 3 used equipment as incentive or motivation during the walking test; 1 assessed children and adults and 13 were duplicated. Manual search found 2 references.

**Fig. 1:**
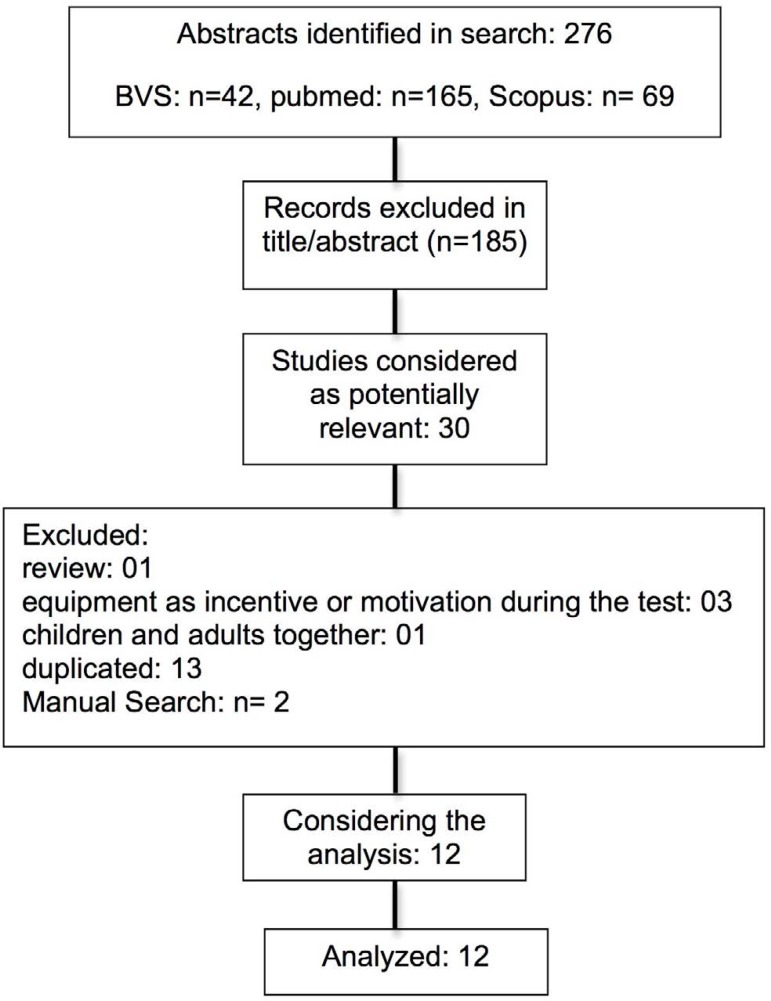
Diagram flow of studies in this review.

Twelve studies matched the inclusion criteria and were fully analyzed and approved by both reviewers. Figure 1 shows the PRISMA flow diagram of studies in this review. The reference list of the included studies did not show additional relevant studies.

### Quality of the 6MWT

The majority of the selected studies matched the ATS guidelines for the 6MWT (Table 2).

**Table 2:** Characteristics of the selected studies.

Study	Sample			Location		Encouragement			
	n	Age ranged (years)	BMI (kg/m^2^)	Flat/straight corridor. Hard surface	Corridor length (m)	Standardized phrases	ATS guidelines	Same technician	
Li et al.^[17]^	1445	7 to 16	18.4 ±3.4	Yes	30	Yes	Yes	Yes	
Kanburoglu et al.^[24]^	949	11 to 18	22.47±2.7	Yes	30	Yes	Yes	Yes	
Lammers et al.^[20]^	328	4 to 11	16.9 ±2.6	Yes	30 to 50	Yes	Yes	Yes	
Priesnitz et al.^[22]^	188	6 to 12	18.5±3	Yes	30	Yes	Yes		
Saad et al.^[18]^	200	6 to 16		Yes	40	Yes	Yes	Yes	
Klepper & Muir^[14]^	100	7 to 11	18.5±6.5	Yes	15 to 25	Yes	Yes (but It was not said that they could stop and rest)	Not informed	
Tonklang et al.^[16]^	739	9 to 12	Not informed	Yes	30	Yes	Yes	No	
D’Silva et al.^[25]^	400	7 to 12	14.7±0.7	Yes	30	Yes	Yes	Yes	
Goemans et al.^[19]^	442	5 to 12	Not informed	Yes	25	Yes	No	No	
Ulrich et al.^[21]^	496	5 to 17	17.9±3.3	Yes	30	No	Yes	Not informed	
Gatica et al.^[23]^	192	6 to 14	19.49±1.83	Yes	30	Yes	Yes	Not informed	
Rahman & Alnegimshi^[15]^	136	6 to 11	16.65±1.75	Yes	30	Yes	Yes	Yes	

### Study Characteristics

From the 12 studies, 11 were written in English and one in Spanish. The reference values for the 6MWT covered 12 different countries: China^[17]^, United Kingdom^[20]^, Tunisia^[18]^, Chile^[23]^, Turkish^[24]^, United States of America^[14]^, Thailand^[16]^, India^[25]^, Belgium^[19]^, Switzerland^[21]^, Saudi Arabia^[15]^ and Brazil^[22]^ (Table 2). The final sample of the selected studies ranged from 100^[14]^ to 1445^[17]^ children/adolescents, and age of participants ranged from 4^[20]^ to 18^[24]^ years old. Ten of 12 studies included both genders. One study only included boys^[19]^ and another one only girls^[15]^ (Table 2).

All the included studies used a convenience sample size that was partially or totally recruited from schools. Six (50%) studies reported the use of randomization for sample selection^[15-17,19,24,25]^.

### Length of the Corridor

All studies performed the 6MWT indoors, along a flat and straight corridor with a hard surface following the ATS guidelines. Among the studies, the corridor length ranged from 15 to 50 meters^[14,20]^. Most of the studies used a corridor of 30 meters^[15-17,21,23-25]^, 2 studies used a corridor from 40 to 50 meters^[18,11]^ and 2 from 15 to 25 meters^[14,19]^.

### ATS Guidelines Confrontation

Seven from 12 (58%) studies described a pretest rest period (10 minutes) and 8 (67%) studies marked the turnaround points with a cone^[15-18,20,23,25]^. All studies used the test instructions to participants outlined in the ATS guidelines. Participants were instructed to walk as fast as possible without running or jogging being allowed to stop. Researchers encouraged the participants with standardized phrases (Table 2).

### Number of Tests

More than half of the searched studies in our systematic review performed a unique test^[15-17,19-21,23-25]^, most of them in a 30 meters corridor^[15-17,19-21,23-25]^. Three studies performed 2 different tests with a 15, 30 or 60 min of interval^[14,18,22]^ (Table 2). Two studies used the best walked distance to establish the reference value, although the authors had reported statistical difference between them^[14,18]^. Although no statistical difference among different corridors in the studies, volunteers walked longer distances on the 30 meters corridor^[16,17,25]^.

### Walked Distance and Reference Equations (Variables Influencing the Walked Distance).

Of the 12 included studies, 7 (58%) reported descriptive data and 6 (50%) formulated reference equation for the walked distance in the 6MWT^[17-19,22,23]^. Considering the 6 studies that established the reference equations, 2 established specific equations for males and females^[17,21]^. Hence, we have 7 available equations in the literature to predict the walked distance in the 6MWT. In most studies, the reference equations were obtained by using linear multiple regression models, including demographic and anthropometric features as independent variables (Table 3). The prevalence of the variables associated with the walked distance was: height (100%), heart rate (80%), age (70%) and weight (60%).

**Table 3:** Standard data extraction from methodologies of reported 6MWT studies in healthy children.

Study	Age ranged (years)	Sample	BMI (kg/_m2_)	ATS recommendations	Length walking course (m)	Side effects (harms)	Number of performed tests	6MWD (m)
Li et al.^[17]^	7 to 16	1445 (805 boys)	18.4±3.4	Standardized instructions and encouragement	30	None	1	664±65.3
Lammers et al. ^[20]^	4 to 11	328 (178 boys)	16.9±2.6	Standardized instructions and encouragement	30 to 50	None	1	470±59
Priesnitz et al.^[22]^	6 to 12	188 (96 girls)	18.5 ±3.0	First of two tests used standardized instructions and encouragement	30	None	2 (interval of 30 min)	579.4 ±68.1 (1 test) 569.2±83.4 (2 test)
Saad et al.^[18]^	6 to 16	200 (100 boys)	Not informed	Best of two tests. standardized instructions and encouragement at the 2^nd^ test	40	None	2 (interval of 60 min)	694±72 (girls) 707±102 (boys)
Klepper & Muir^[14]^	7 to 11	100 (57 girls)	18.5±6.5	Two tests. Standardized instructions and encouragement	15 to 25	None	2 (interval of 15 min)	518.5±72.5
Tonklang et al.^[16]^	9 to 12	739 (403 boys)	Not informed	Standardized instructions and encouragement	30	None	1	677±62.2
D’Silva et al.^[25]^	7 to 12	400 (202 boys)	14.7±0.7	Standardized instructions and encouragement	30	None	1	609±166
Goemans et al.^[19]^	5 to 12	442 boys	Not informed	According to McDonald et al.	25	None	1	582.2±88.2
Ulrich et al.^[21 ]^	5 to 17	496 (252 girls)	17.9±3.3	Standardized instructions with no encouragement	30	None	1	618±79
Rahman & Alnegimshi^[15]^	6 to 11	136 girls	16.65±1.75	Standardized instructions and encouragement	30	None	1	595.7±61.35
Kanburoglu et al.^[24]^	12 to 18	1045 (506 boys)	21.19±3.15	Standardized instructions and encouragement	30	None	1	542±97 (boys) 530±92 (girls)
Gatica et al.^[23]^	6 to 14	192 (100 girls)	17.55±1.26	Standardized instructions and encouragement	30	None	1	596.5±57 (girls) 625±59.7 (boys)

The study by Tonklang et al.^[16]^, performed in Thailand, showed the highest walked distance in the 6MWT (677±67 meters, using corridors between 15 and 25 meters). On the other hand, the study by Klepper et al.^[14]^, performed in the United States of America, showed the lowest walked distance (518±73 meters, using corridors of 30 meters) (Table 2). The walked distance ranged 159 meters between these studies. The variable sex influenced the distance, being higher in men than in women^[14,16]^.

### Side Effects

None of the studies reported any side effect related to the 6MWT (Table 2). The 6MWT is a very safe method to assess exercise capacity in healthy children and adolescents.

## DISCUSSION

The main findings of this systematic review showed that the reference value for the 6MWT ranged up to 159 meters. The walked distance was higher in Thailand^[16]^ and lower in the United States of America^[14]^. The most prevalent variables in the reference equations were height (100%), heart rate (80%), age (70%) and weight (60%). The majority of the studies performed the 6MWT according to the ATS guidelines.

Although there are systematic reviews about the 6MWT, none aimed to compare the walked distances and reference equations for healthy children/adolescents of different nationalities^[14-25]^. The importance of our review is to warn clinicians and researchers about the differences of the reference values for the 6MWT found in the literature. Caution is needed when using a foreign reference value for the interpretation of a 6MWT in children/adolescents. Our systematic review clearly showed that the reference value for the walked distance can vary up to 159 meters, which is of great clinical importance if we consider the minimally significant difference already established in several adult populations, such as 32 meters for heart failure^[29]^, 25 meters for coronary artery disease^[30]^ and 30 meters for chronic pulmonary obstructive disease^[31]^. Unfortunately, no minimally significant difference is available for children and adolescents.

Despite the wide range of the maximum walking distance, none of the studies outlined the socioeconomic profile of the participants. A curious fact is that the highest walked distance was obtained in a developing country (Thailand)^[16]^ and the lowest in a developed country (United States of America)^[14]^. Nevertheless, the corridor length used in Thailand was lower than that used in the United States of America, what can underestimate the maximal walked distance.

From a methodological point of view, the studies used random and multicentric samples, but no data of sample size calculation was available in the studies. In addition, the studies did not report the importance of including centers in other regions of the country itself, which could contribute to more consistent establishment of reference values in countries with large territory, such as Brazil.

The most prevalent variables in the reference equations were height, heart rate, age and weight. This prevalence was not surprising because they are well known to be associated with exercise performance. In general, taller individuals tend to have longer leg length and consequently wider last^[19]^. The behavior of the heart rate has been associated with an increased physical performance, since the lower resting heart rate usually reflects a greater prevalence of the parasympathetic nervous system and higher fitness^[32]^. It is also well known that oldest children and adolescents have better exercise performance than youngest ones^[20]^. This may be a reflection of greater stature and greater influence of anabolic hormones throughout the growth^[33]^. In adults, we know that exercise capacity can decline from 8% to 10% per decade in both sedentary and athletic populations^[34]^. Just as in adults, it is known that children and adolescents with higher weight have lower exercise capacity than the ones with normal weight^[35]^.

Except for one study, the reliability of the 6MWT reference equation was investigated comparing the predicted distance to the measured distance. Studies that performed just one test considered this information as study limitation, once the learning effect can happen^[2]^.

The use of the reference values for the 6MWT brings a more precise interpretation of this test in clinical practice and research. However, health professionals from countries that do not have reference values for the 6MWT should be aware about the selection of a reference value established in another country. Otherwise, the test interpretation can be compromised.

Our systematic review has limitations. First, there is no wellestablished tool to assess risk of bias for studies that aimed to investigate reference values. Second, it was not possible to analyze the reference values for children and adolescents separately.

We suggest for future research the use of standardized corridor length according to new guidelines for the 6MWT, *i.e*., at least 30 meters. Furthermore, it is important to have a sample size calculation and distribute the sample in different regions of the country, especially for those with large territory. Authors should also provide reference equation for their population.

## CONCLUSION

The reference value for the 6MWT in children and adolescents ranged substantially from studies in different countries. A reference equation was not provided in all studies, but the ones available took into account well established variables in the context of exercise performance, such as height, heart rate, age and weight. Countries that did not established reference values for the 6MWT should be encouraged to do because it would help their clinicians and researchers have a more precise interpretation of the test.

**Table:** 

Authors’ roles & responsibilities	
LAPC	Realization of operations and/or trials; final manuscript approval
VJSF	Conception and design study; final manuscript approval
LGM	Manuscript redaction or critical review of its content; final manuscript approval
MGN	Manuscript redaction or critical review of its content; Final manuscript approval
MF	Conception and design study; final manuscript approval
VOC	Realization of operations and/or trials; final manuscript approval
